# Towards a Relational Phenomenology of Violence

**DOI:** 10.1007/s10746-013-9269-x

**Published:** 2013-03-07

**Authors:** Michael Staudigl

**Affiliations:** Vienna, Austria

**Keywords:** Phenomenology, Violence, Embodiment, Symbolicity, Experience, Relationality, Slapping

## Abstract

This article elaborates a relational phenomenology of violence. Firstly, it explores the constitution of all sense in its intrinsic relation with our embodiment and intercorporality. Secondly, it shows how this relational conception of sense and constitution paves the path for an integrative understanding of the bodily and symbolic constituents of violence. Thirdly, the author addresses the overall consequences of these reflections, thereby identifying the main characteristics of a relational phenomenology of violence. In the final part, the paper provides an exemplification of the outlined conception with regard to a concrete phenomenon of violence, i.e., slapping, and a concluding reflection upon its overall significance for research on violence.

## Introduction: Violence and Sense

Two main obstructions tend to hamper research on violence. The first revolves around the concept of violence and the danger posed by an essentialization of the phenomenon that is *determinative* of its *sense*, that is, it views violence exclusively in terms of response (as counter-violence), resource (as an instrument), or structural or pathological predispositions (Wieviorka 2003: 42f.). The second obstacle consists of the opposite view, namely to deem violence that escapes these patterns of interpretation *senseless* (see Best 2000; critically Blok 2000; Mamdani 2004: 3f.); and it is exactly this view that quickly leads to an all too violent sidelining. In my view, these two problems overlap in a deeply problematic understanding of the *relationship between violence and sense* that reduces violence to an instrumental action of a “strong subject” but largely overlooks its silently functioning poietics and socio-technological dimensions.

This relationship has indeed been neglected all too often in favor of an analysis of the *causes* of violence—be it sociological, psychological or anthropological.^1^ While such analyses were carried out based on a seemingly consolidated idea of *what violence is*—that is, “the deliberate infliction of bodily harm”—such a definition fails to take into account *how violence is experienced*; the “sense” of it according to its “protagonists”.

In response to such a reductive approach, it has recently been suggested that a theory of violence be developed that puts the *subject at its center*.^2^ Subject-centric approaches indeed tend to examine the experience of a “loss of sense,” as undergone by the *recipient* of violence (see Mensch 2009: 72–80, Dodd 2009), a (not necessarily instrumental) “gain of sense,” as experienced by its *actors* (see Sutterlüty 2003: 77–110; Wieviorka 2007: 111–130), or of the duty to “articulate sense” attributed to “third persons,” e.g., witnesses and bystanders, judges and therapists.^3^ From a phenomenological standpoint, much effort has been expended in the development of a phenomenology of violence *suffered* (see Delhom 2000). In this context, violence is analyzed in terms of being “destructive of sense” (Mensch 2009: 72), that is, as a break in our inter-subjective processes of making sense together (cf. Wieviorka 2003: 42f.). Yet the question remains whether such a perspectival approach is really sufficient. Is it successful in bringing the very phenomenon of violence to the fore? Does it, finally, bring the full potential of phenomenology to bear?

As I want to show in this article, it is indeed of utmost importance to examine the various faces of violence in their intrinsic *relationality*. To unveil their relational character, I will attempt to substantially broaden the phenomenological concept of sense. By *sense*, I propose not only to examine the immanent accomplishments of the subject’s engagement in and with the world, but, first and foremost, a relation that unfolds in-between the one and the other. Sense, in other words, unfolds in the subject’s relation with those it encounters in this world, who can make this world appear to it, *dys*appear, or, finally, disappear, and accordingly shape its self-understanding, self-conception, and agency.

The discussion of violence in terms of a relational phenomenon or inter-phenomenon requires emphasis on two matters in particular: firstly, that the lived sense of violence cannot be extracted from just *one* perspective or viewed against the background of an unshakeable “reciprocity of perspectives” (Schutz), a foundational (e.g., cosmological) order, a teleological order (epitomized by reason’s historical tendency to self-realization), or a procedural (e.g., legal) order. This insight rather calls upon us to consider those dimensions of our inter-corporeal existence in which the lived sense of violence develops in manifold ways without our active participation. Secondly, the discussion of violence as a relational phenomenon is testament to the fact that we have grown used to understand violence as an *exception* to our intrinsic sociality (or, at the very least, sociability) and communicative competence. This view is a result of the tacit assumption that those events to which no (social) sense can be attributed are simply divorced from any premise of cultural activity (Koschorke 2008: 322).^4^ This assumption and the resulting “abhorrence” (*Perhorreszierung*) of senselessness is by no means unproblematic: within it, there lies a rather potent *metaphysics of the social*, for which “sense”—echoing Luhmann—is in the last instance an “undeniable, differenceless category” (Luhmann 1984: 96). Only when one recognizes that violence, as an exemplary phenomenon of “negative sociality” (see Hetzel/Liebsch/Sepp 2011), is not only (though always) destructive in character, but also and always the performer of poietic or socio-technological functions, whose subjective powers of sense-bestowal and, more importantly, powers of social formation cannot be ignored,^5^ does one begin to approach a comprehensive understanding of violence. When couched in alternate terminology, subjectification and desubjectification are inextricably linked to one another in violence (see Wieviorka 2007: 111f.).^6^

Any theoretical examination of violence that takes this matter seriously and in its multiply irreducible meaning for human life forms must tackle the problematic relation of sense and violence. In doing this, such an examination recognizes, on the one hand, that the irreversibility of violence condemns us to a “life under and with violence” (Liebsch 2007: 35f.). This is not only the case because violence represents a factual and *optional* act, but, rather, because the social orders in which we move, perceive, and act are themselves *violent*. This violence of irretrievably contingent orders is in no sense a sign of their disfunctionality—rather it is essentially constitutive of them, since orders function selectively and exclusively (see Waldenfels 1996). A failure to recognize this intrinsic affinity between orders and violence would result in a theoretical repetition of the contrast between “order” and “disorder” that habitually pre-structures our perceptions, interpretations, and actions without considering their constitutive inter-dependence. But what thereby remains underexposed or, better, bracketed is the fundamental fact that orders always (re)produce “disorder,” i.e., always—in the sense of an “implant of fear” (Reemtsma 1996) or of the “threat of the unruly” (Sheth 2009)—include it by excluding it, since an order must always anticipate the possibilities of its transgression, invasion, subversion, or destruction in order to uphold itself.^7^

Insofar as our life-worlds are permeated and constituted by “ineliminable violence,” the finding of a “final solution” for violence cannot settle the matter. Rather, such a gesture is testament to the self-righteousness of a brand of reason that denies its own violence, that proclaims such violence to be a legitimate legal institution (“legitimation via procedure”) or that announces its elimination through the facticity of power relations, which others in turn condemn for their illegitimacy.^8^ But this separation of legitimacy and illegitimacy is a specific historical product of a genesis that tends to forget itself in its consequences, bracketing its myth from the process of historical becoming and critical reflection. Since there are no “final solutions” to violence that are not themselves violent, since violence is also intrinsic to, e.g., law (see, in particular, Derrida 1992), a *critique of violence* must not intend to offer more than the “quest for spaces of lesser violence” (see Liebsch 2007).

In order to move this undertaking forward, it is first necessary to examine the *different faces of violence* in phenomenological fashion. The task is therefore to analyze the often subtle ways—beyond the physically tangible or visible—in which violence makes its way into human life forms, shaping them and settling in these, oftentimes undetected. To some extent, this would mean to stop the above-mentioned quest for space from itself proceeding in an unreflected, violent manner as a result of preliminary decision on what counts as violence. To a further extent, this would mean to show how different forms of violence motivate one another and play into each other’s hands. Therefore, they must be understood in relation to each other.

This article provides a first sketch of *such a relational phenomenology of violence*. In order to do this, I shall first outline the basic structure of the phenomenological method I deem most adequate to tackle phenomena of violence (1.). Given that violence *exists* (but not violence *tout court*) and that it must always be considered within the horizon of its particular orders, I shall then analyze the double “fact of violence,” its affective and symbolic dimensions and their inextricable interlacement (2.). In a third part I shall address the consequences of these reflections, thereby identifying the main characteristics of the relational phenomenology of violence that is to result therefrom (3.). Finally, I will close with a reflection concerning possible applications of the outlined conception and some future lines of argumentation.

## The Phenomenological Method and an Analysis of Violence

In my opinion, the difficulties described above in determining the sense of violence can be overcome through the development of a *more extensive notion of sense*. The scope of this notion far surpasses the strict boundaries of linguistic meaning or signification. Seen phenomenologically, sense is not only that which can be expressed or permanently recorded. Moreover, it cannot be explained in the sense of a teleology of instrumental or normative sequences of action. Husserl points this out in his *Ideas*, in which he “expanded” the notion of sense, so that it could include the intentional directedness of all conscious acts (Husserl 1982: 285; see Tengelyi 2007: 5ff.).

The overarching aim of phenomenology is to elevate the intentional horizons of the experiential life, in which we move, think, and act on an everyday basis, into the thematic without these actions being mainly and most often thematic. It asks how sense is *constituted for* the subject in its experiential life, that is, how the subject *bestows sense on the given or fails to do so* (not merely as a result of Marcel’s mysteries of existence, but also given the bare resistance of things or the traumatic consequences of experiencing violence) and how it at once *constitutes itself* in this confrontation with the facticity of the given.^9^

In his later “genetic phenomenology,” Husserl also expanded this intentional notion of sense. Apart from acts of subjective “sense bestowal” (*Sinngebung*), he also begins to consider processes of passive, especially bodily “sense formation” (*Sinnbildung*) and historic events of “symbolic institution,” (*Sinnstiftung*) that can no longer be understood as noematic correlates of a pure ego’s noetic activities. This allowed Husserl to tackle a previously unseen dynamic of subjective sense and, further, its inter- and trans-subjective constitution. As was becoming increasingly clear to him, though without being able to spell out the consequences of this insight in a systematic fashion, the meaningful constitution of the given is in no way exhausted by an explication of the active “sense-bestowing” acts of the subject. Rather, in his “genetic phenomenology,” Husserl sought to trace the passivity and anonymity involved in all subjective sense (*Sinn*), in the way it *forms and unfolds itself* without our active intervention. From a “generative perspective” he finally considered its *historicity* and *alterity*, which appear to run counter to our intentions, possibly distorting these.^10^

Thus viewed, Husserl’s original idea of a “constitutional analysis” turns more and more into an inquiry into the *foundational interlacement of subjective acts of sense*-*bestowal, anonymous processes of inter*-*subjective sense*-*formation*, and *symbolic institutions of trans*-*subjective meaning* (which can also be understood as patterns of sense *construction* in sociological terminology). Given this interlacement, the “autochthonous significance of the world” is related both to “the dealings which our incarnate existence has with it, and which provides the ground of every deliberate *Sinngebung*” (Merleau-Ponty 2002: 512), and to its generatively derived symbolic articulations.^11^ This fundamental expansion of the notion of constitution has decisive consequences for the phenomenological understanding of the *subject*. Taking this foundational interlacement of three pillars of sense into account, I propose to read the self-constitution of the subject as the constitution *of* the other in the twofold sense of this genitive. Hence, considered as a relational concept, sense can neither be reduced to a subject’s activities, nor to anonymous processes of signification. Rather, the “subjective side” of the “correlational a priori” is itself dependent upon the process-like quality of the appearing world. Thus, it is a largely passive *process*. Its integrity only forms and develops as a result of the openness of intentional life for the appearing. In the last analysis, this openness is due to our *embodiment*. Since its identity follows from its spontaneous participation and its passive involvement in the process of appearing, that is, from the fact that it appears to itself in the articulation of the space of appearance (i.e., in the “coherent deformation” of pre-given sense), I propose to rethink the intentional subject as a *performative self*.

Given this comprehensive, relational conception of constitution and self-constitution, it should become clear why I deem phenomenology to be appropriate for an examination not only of various phenomena of violence, but also of the *very phenomenon of violence*. This is because it enables us to unveil pre-given structures of meaning (e.g., judgments) in their intentional “sense-genesis” and “essentially necessary sense-history” (cf. Husserl 1969: 207f.), which is largely unavailable to us, yet in which, for us, *experience achieves its meaningful constitution*. In our context, this is important because it allows us to address the phenomenon of violence *without having decided on its social sense or non*-*sense, i.e., in terms of rationality or irrationality*. In showing that *there is sense*, but not sense *tout court*; in showing that sense *develops* without being wholly attributable to an autonomously constituting subject or, conversely, to objective orders or to the effects of structures and discourses, i.e., in showing that subjectivity is required for meaningful connections to arise factually, phenomenology warns us against taking the presumption of given sense to be sense itself. For this would mean nothing else than to hold a contingent attitude towards the “things themselves” to be absolute, be it a “cultural self-understanding” (i.e., Scheler’s “relative-natural worldview”), be it the scientific attitude (i.e., Husserl’s “naturalistic” or “objectivist attitude”), or a specific (*sonderweltlich*) attitude (e.g., the “legal attitude”). Rather, phenomenology insists upon the bracketing of such reductive attributions of sense, in order to emphasize their emergence in experience and to examine whether any such evidence is indeed relevant.

In the *Crisis*, Husserl uses this process of inquiry to criticize the bottomlessness of the objective sciences. As regards the matter at hand, such an inquiry does not entail an *epoché* of the substruction of an objectively true being, but an “ethical epoché” (Husserl 1959: 319; see Gilligan 1997: 91–94; Ò’Murchadha 2006: 10–11). This allows us to sideline the “hunt for causation” (Whitehead 2004: 55) regarding violence and to withdraw from the apparently inevitable discourses of justification, which tend to overshadow their own limits and violence (see Waldenfels 1991). That is to say that we shall not address violence phenomenologically in terms of its illegitimacy, irrationality, or amorality within a given order, i.e., in terms of a moral economy, but purely in its subject-relative, experiential sense. This, however, does not imply that we will find ourselves in a “neutral zone of indifference,” in which anything and everything ‘goes’. Rather, the “ethical epoché” seeks to approach the “wild” space of experience that becomes visible where the taken-for-grantedness of factual normative orders has turned brittle or collapses (which is the case with violence in particular). In this pre-normative (though not lawless) space, one is confronted with the claims of the other, which are not valid in a legal sense, but confront us with her unavoidable “ethical appeal” (Waldenfels 2003: 141). As experiential *excesses* that run counter to our will, they do not allow us to simply turn away and to return to the everyday state of things with sanctioned moralities that tell us how to cope with whatever happens. It is only possible for others to shake this taken-for-grantedness of given normative orders and to *appeal* to us in this basal, pre-linguistic and pre-legal sense because we primordially do not encounter them in moral, legal, or, generally viewed, discursive terms, but on the pre-reflective basis of an inter-corporeal or “inter-kinesthetic affectivity” (Behnke 2008).

Hence, the decisive horizon within which we shall examine violence does not purport to be a universally valid normative framework, i.e., one that would allow for a definitive decision on the existence of violence. Rather, it is the *affective horizon* of our embodied “sensibility” for the vulnerability of the other (see Liebsch 2008). Only the recourse to this “knowledge” derived *from the singular appeal of the other*, i.e., one that is not based on a normative order or “lived moral conditions,” will enable us to correct the tendency to see violence as a judicially over-determined phenomenon, i.e., as *per se* illegitimate and, in the final analysis, “senseless”. On the contrary, this step will enable us to analyze it as an intrinsically meaningful event that holds all those involved under its fatal spell. Only by recourse to the level of our inter-corporeal existence, on which the appeals of the other are neither leveled out positivistically nor over-determined moralistically, but addressed in terms of an *ethos in statu nascendi*, it can be understood how these appeals may be silenced, as a result of which violence against the other (in spite of her ethical resistance) becomes *possible* in the first place.^12^

Viewed against this background, I propose to rethink violence in terms of vulnerability (both the other’s and our own). A *refusal to respond* to the appeal of the other is to *violate* her, i.e., her embodied being and/or the claims that make up her self-referential integrity. In other words, not only physically injurious acts of violence can, therefore, be *violating*. But what exactly does this mean? Does this expansion (which was already at work in our concept of the violent character of orders) blur the very concept of violence in a counterproductive way? As regards my hypothesis, this is not the case. To consider violence in terms of violation requires to rethink it beyond its normative over-determinations, i.e., to rethink it *starting from the other* and the *affective order* she establishes.

## The Double “Fact of Violence”

In order to substantiate this broadened concept of violence, a change of focus is required. Instead of focusing on (intended) *injuries*, I propose to focus on the ways we deal with manifold instances of *vulnerability*. After all, the many faces of violence can only be understood in the light of our vulnerability, of its inter-subjective articulations, and cultural as well as political determinations. This perspective ought to show that, as regards violence, our *ability to form sense inter*-*subjectively* is at stake, that is, our *capacity to primordially articulate alien sense*. This is true for *violence suffered*, which must be endured in its destructive power and meaningfully assimilated; it is also true for *violence exerted*, in which the basic responsive infrastructure of inter-subjective sense-formation is interrupted, suspended, and possibly collapsed; and it is finally also true for *violence witnessed*, in which the articulation of the violation of others becomes the duty and responsibility of a “third party”.

In each of these different perspectives on violence, we therefore come up against the key experience of *vulnerability* in a different way: as undeniably suffered, as indifferently exploited, or as perceived and dealt with by “third parties”. The phenomenal core of the different faces of violence is thus undeniably its relation to the fact of our generic vulnerability. Being vulnerable, being able to indifferently exploit the other’s vulnerability, and having a basic sensibility for the vulnerability of others are thus to be taken as different and yet related parts of the same phenomenon, namely of our irreducible exposure to the irreducible fact of our vulnerability. In order to show what exactly is at stake when focusing on vulnerability, I will now delineate the full scope of this concept, which is of key importance for a relational and, hence, integrative understanding of the many faces of violence.

As to my hypothesis, not only the subject’s *objective body and its integrity* is the site of her vulnerability. Violence can equally—and often does just this—affect such contexts of meaning, in which the lived self-understanding of the subject, that is, her comprehensive self-referentiality or, traditionally put, her *personal identity*, is *embodied*. In contrast to the enduring preference for direct or physical violence in the social sciences, this multifarious vulnerability of the subject goes far beyond the mere metaphorical tool. And yet the viability of the concept of violence here proposed is far from self-explanatory: it largely depends on a shift in emphasis to the *lived integrity of the subject on the level of her phenomenal body* and the existential vulnerability of the person and her social identity.

This shift in paradigm must be performed by means of a phenomenological theory of lived embodiment. On this topic, Merleau-Ponty states that “(in as much) as we are incarnate beings, violence is our lot” (Merleau-Ponty 1969: 109). This declaration is valid not only for the “recipients” but also for the “actors” of violence and finally for those who experience it from a “third position,” but who, on account of their bodily and affective presence, can never completely remain “unconcerned spectators”. As the preceding reflections have shown, the shared fact of our incarnation—or, to be more precise, the primordial fact of our “intercorporeality” (Merleau-Ponty 1968: 141–143; see Weiss 1999)—accounts for both our vulnerability and our agency, just as our sensitivity and indifference vis-à-vis others remain to be understood in light of its social articulation. Hence, violence must be thought fundamentally in relation to our lived embodiment and affectivity. In other words, no matter how violence affects our self-relational integrity and/or the claims rooted therein (for instance, the claims to political recognition as a morally assignable being or to integration in a context of social self-realization), our embodied existence functions as the ultimate point of reference for all violence (see Srubar 2013).^13^

In this context the substantiation of the above-mentioned comprehensive concept of violence gains its validity. Thus, through the “intentional overreaching” (Husserl 1960: 112) on the part of the embodied subject onto the level of habituality and ideality, which can be described as a lived chiasm of embodiment and expression, i.e., in terms of *symbolic institution*, the phenomenal domain of violence extends beyond its physical and visible forms of appearance. Hence it is not only “in my body as I immediately possess it” that I experience “violence done to me” (Hegel 1995: 111f.). Rather, being embodied and, thus, being open to the “transphenomenal cognitive process” of institution (Merleau-Ponty 2010: 58) means that violations and the experience of violence are *mediated* by the body and its symbolic-semantic shaping regardless of their origin, physical or not. I am therefore vulnerable in terms of what Merleau-Ponty calls our “habitual body” (Merleau-Ponty 2002: 95, 101). Here, it is a matter of those ideal contexts of meaning, which make up the habitualized—and thus potentially reactivatable—scope of my primordial “I can” and which Judith Butler has recently called “the socially ecstatic structure of the body” (Butler 2009: 33). Among these ideal embodiments, “symbolic institutions,” or “self-extensions”^14^ of my existence, the “body of speech” in Merleau-Ponty, Levinas’ “dwelling,” what Bourdieu calls the “cultural field” and, last but not least, the “home-world” in Husserl can be counted: it is their meaning-generative practical intelligibility that is the gateway for different forms of indirect, e.g., linguistic, symbolic, structural, or cultural violence.^15^

As already pointed out above, “constitution” implies a relational event. It implies the appropriation of the world as a context of sense in perception, thought, action, interaction and, correlatively, in the constitution of our own selves. Embodiment, self- and sense-constitution are therefore intrinsically interrelated. They make up an indissoluble and reciprocal foundational context. When any one of these levels is violently attacked, they are always all affected (see Mensch 2009: 75f.). In this way, *physical* violence, for instance, not only affects the objective body, but also the lifeworldly idealizations of our “I can” and therefore our habitual openness to the world. Conversely, *psychological* (e.g., verbal) violence always has an effect on our bodily movement in our surrounding social world and on our “orientation” in it (see Ahmed 2007). *Structural* violence, on the other hand, interferes in the habit formation of the subject, thereby creating “docile bodies” that silently accept the discriminating social (e.g., legal) structures that shape them and their possibilities of (self-)perception, interpretation, and action (see Bourdieu 2002: 33–36). As for *cultural* violence, it attacks the “collective bodily existence” (Husserl 2008: 181) of the subject, i.e., the silently shared patterns of its inter-corporeal existence alongside which the individual “I can” primordially develops and realizes itself in a pre-given cultural nexus. Thus, put slightly differently, different forms of violence attack the different ways in which we realize and understand ourselves as irreducibly embodied beings.

Viewed against this background, the “fact of violence” turns out to be a *constitutively twofold facticity, that is, both affective/bodily* and *symbolic/meaningful*. On the one hand, the excess of affectivity that follows the *experience of violence* (through the pain caused by violation) also makes us experience extraordinary attributions of sense, such as the destruction of derived social meanings. Conversely, the imposition of extraordinary attributions of sense can cause an affective excess. On the other hand, in the *exercise of violence*, an affective deficit in sensibility towards the vulnerability of the other leads to forms of sense-bestowal that neutralize, overlook, and finally negate the other’s ethical appeal and the singularity that this entails.

This overlap of affectivity and symbolicity—or, to be more precise, of the affective power of sense-bestowal and the symbolic power of affectivity—is constitutive of violence in all its shapes and forms. Put differently, there is neither a pure physical violence—since it is always already symbolically over-determined—nor is there a purely symbolic violence, i.e., one that would not softly and silently be engrafted onto our bodies in the ‘back of one’s consciousness’.

Whereas I turned to the phenomenology of embodiment to show that our embodied condition functions as the ultimate reference of all forms of violence, I still need to elucidate the phenomenological concept of “symbolic institution,”^16^ which makes up the second integral part of all violence. To speak of “symbolic institution” is to designate those pre-given intentional horizons of inter-subjective sense-sedimentation that allow us to *articulate* our experiences as socially meaningful events. By institution, basing himself on the late Husserl, Merleau-Ponty means first and foremost anonymous “events in an experience which endow the experience with durable dimensions, in relation to which a whole series of other experiences will make sense, will form a thinkable sequel or a history” (Merleau-Ponty 2010: 77). This notion therefore describes the processes of acquisition, sedimentation, and possible reactivation of inter-subjectively shared faculties of sense-making. In this context, the reactivation of instituted meaning structures assures the continuity and coherence of the experience without, in the face of the contingency of the given, drifting off into an endless process of verification and modalization. As Merleau-Ponty showed, institutions do not provide us with *concepts* or interpretive devices, but open up to us historically derived *styles of being*. Therefore, this concept not only allows for a description of “intention[s] without subject” that “happen beyond what is willed, experienced, known” (as in the case of life’s organismic fecundity, the drama of puberty, or the inter-corporeal dynamics of emotion) (2010: 6, 10), but also for an analysis of those (public) institutions (language, the field of culture, works of art) that enable us to move *in* ideal contexts of sense and *between* “finite provinces of meaning” (such as religion, science, and politics). Essential to our discussion is the fact that by way of our habitual usage and practical incorporation of such institutions their ordering power becomes a constitutive, yet largely pre-reflective part of our existence.^17^

We see better now, how “symbolic apparatuses”—using again a Merleau-Pontyian term—incorporate the power to institute sense. This sense, however, escapes the paradigmatic form of constitution as exposed by Husserl in his orientation on a phenomenology of perception; this sense can therefore not be retrieved by simple recourse to the purported immediacy of intentional experience. Against the idea of a rectilinear foundation of expression, signification, and, finally, institution on the objectifying intentionality of perception, the later Merleau-Ponty demonstrated that “the originating” is not at all unitary, but that it “breaks up” (Merleau-Ponty 1968: 124). Marc Richir takes up this insight and proposes to speak of a “symbolic creation of sense” (Richir 1996: 333). The ordering registers of “symbolic institution” are therefore not just to be taken as a “medium” that “mirrors” foundational intentional processes.^18^ Rather, we have to see them as “symbolic matrices” (Merleau-Ponty 1968: 240) that incorporate basic patterns of a given “cultural field”. This not only implies that every social or cultural act (particularly violence) is always already symbolically over-determined (see Merleau-Ponty 1973: 200, 2010: 7). It also means that violence itself also interferes with the registers of symbolic institution and that it can be carried out through them: since they are of paramount importance for the self-constitution of the subject, their destruction reciprocally affects its selfhood.

The concept of the “symbolic matrix,” taken as the institution of the “openness of a field, of a future according to certain dimensions, [and of] the possibility of a common adventure and of a history as consciousness,” (Merleau-Ponty 2010: 13) designates the establishment of a trans-subjective field of “inter-individual knowledge” (2010: 58). Thus viewed, it allows us to understand why we are open and vulnerable to ephemeral forms of violence, such as verbal, symbolic, or structural violence. Though the “socially ecstatic structure of the body” (Butler 2009: 33) is *dependent* upon our embodied existence, this is the case because its symbolic articulations provide us the freedom to exist independently of the “pragmatic motive” (Schutz 1962: 208f., 1970: 142) that permeates our everyday existence. Put differently, these articulations enable us to transcend our need-based being-in-the-world, i.e., to symbolically move, establish, and realize ourselves in a variety of “multiple realities” or “finite provinces of meaning”. Since their reactivation and transformation makes up an integral part of our self-understanding and self-constitution, they become our “second nature” and we *are* vulnerable in them. Their destruction affects our selfhood, but to be kept from accessing them is equally a form of exploiting human vulnerability.

As stated above, we here seek to understand violence in the light of vulnerability. As I have shown in this chapter, violence is not only tied to the physical integrity of our objective body; rather, we are also open to violence in the symbolic articulations and institutions of our existence. After all, these are, echoing Derrida, none other than the “original supplement” of our embodied existence, i.e., not only the “fabric into which all objects are woven” but also the “general system of symbols for the world […] through which we can consequently ‘be at home in’ that world, ‘understand’ it and find significance in it” (Merleau-Ponty 2002: 273, 275). In the next chapter, I will unveil the various implications of this insight into the “twofold facticity of violence” and will show that we need to consider the relational interplay of various forms of violence in order to gain a better understanding of its prevalent forms.

My overall intention is to show that we need to understand violence against the other in relation to the silently imposed and habitualized ways in which we are used to dealing with our own vulnerability.

## Rethinking Violence as a Relational Phenomenon

Recently, Bernhard Waldenfels has proposed to define violence in terms of “addressed adversity” (*adressiertes Widerfahrnis*) (Waldenfels 2003: 149). Given that acts of addressing take place in the “world of human symbols,” (Merleau-Ponty 1973: 200) this definition neatly conjoins the intertwining of embodiment and symbolic institution in the event of violence as I outlined it so far. However, to claim that such an “addressed adversity” is experienced *as violence* is to presuppose two things: firstly, that the *meaning “violence” is attributed* to this social event in relation to a given order and, secondly, that it is founded with *agency*: “Events become deeds or actions by being ascribed to somebody as his or her actions” (Waldenfels 2006: 82).

Attributed *agency* or “*intentionality*,” on the one hand, and the *experience* of suffering and violation, on the other, represent two basic perspectives for the analysis of violence. However, the related theoretical approaches, i.e., the “theory of action” and the “analysis of discourse,” seem to exclude one another:

When we combine the definitional problem of violence as an experiential and endured phenomenon with the problem of violence as an intended act, it seems to come about, on the one hand, through the categorization of the event as violent by a ‘victim’ or witness, without the perpetrator’s intent being relevant to the matter. On the other hand, it appears to come about through the violent intent of a ‘perpetrator,’ whereby the victim need not have deemed the act to be violent. […] I therefore think that ‘violence,’ as a phenomenon, raises a structural difference that can neither be ‘sublated’ in a *figurationssoziologische* way (“figurational sociology”), nor in any other interactionist way. Instead, the question is analytic, that is, *prior to* or *outside* any *moral* opinion of the matter at hand, whereby we must ask whose perspective we would actually (or maybe ‘solely’) like to address […]. (Hitzler 1999: 17; my translation)

The above-mentioned “structural difference” between violence as *intention* and violence as *experience* is, according to Hitzler, constitutive of the “phenomenon ‘violence’”. This point of view is not only leading in the social sciences and humanities; it is also applicable to recent phenomenological debates, as already pointed out in the introduction to this essay. Yet, to agree with it is to beg the question. To do so would be to endorse the dubious splitting of an analysis of violence into “causal research” (be it instrumental or structural) that seeks explanations and leans towards abstract normativity on the one hand and, on the other hand, a “thick description” that all too easily degenerates into an aestheticization or essentialization of violence (see Nedelmann 1997; Trotha 1997). Moreover, this is to deny oneself the possibility to comprehensively examine the very “phenomenon of ‘violence’”. This, however, is the task of the here proposed phenomenological analysis, i.e., by approaching violence as an *event of sense* forged *between* its participants, without its *sense* being exclusively ascribable to their intentions, experiences, or judgments.

But that is not to say that the perspective of *recipients*, for whom the suffering of violence is an *extraordinary* experience from which new sense must be wrested, ought not to be distinguished from the perspective of *actors*, the meaningfulness (*Sinnhaftigkeit*) of which can be denied, but cannot be explained away structural-functionally, socio-biologically, culturalistically, or instrumentally. Finally, there is also the *third party* perspective.^19^ After all, *violence tout court* does not exist: rather, we are confronted with differing experiences and interpretations of social events, which are attributed to or denied the social meaning “violence” against the background of given (i.e., inherently contingent) orders of violence. The *lived sense* of violence, on the contrary, is primordially formed *between* those affected. Thus, it would be ill-conceived to assume that this sense results from their motivated *interactions* or that it could be deduced from *discourses* that structure them (as done by theories of action or theories of discursive construction).

Seen from this perspective, violence, in its *extraordinariness*, must be analyzed as an *inter*-*phenomenon* (*Zwischenphänomen*). With Waldenfels, I take this to be a phenomenon whose “sense” “comes about between me and the other, between us and others, between what is one’s own and what is alien”. At stake here is a sense that, to all effects, “can neither be traced back to the initiative and capacity of individuals or groups, nor to a mediating authority of order or to codified rules” (Waldenfels 2003: 174). Thus, an “inter-phenomenon” is an event in which sense *unfolds* in the interplay of subjective intentions, inter-corporeal processes of sense-formation, and the dynamic “life of institution,” without it being attributable to the sense-bestowing acts of a subject or the structural logic of sedimented “symbolic apparatuses”.

In order to further examine the “inter-phenomenon violence,” we must now examine the two sides of violence, that is, its double—both bodily and symbolic—facticity, in the framework of this relational interplay. This interplay or interlacement of registers indicates that the intention behind violence could not be possible without a predominantly pre-reflexively lived knowledge of the essential vulnerability of the other. It also means that the experience of being violated would not be the experience of violence without the attribution of an “intention”. Lastly, this means that “third parties” would have nothing to testify to (or to ignore) if they were not also previously affected by this vulnerability.

The afore-mentioned dilemma can thus be avoided by moving back to our inter-corporeally lived vulnerability, that is, to the *affective structures of inter*-*subjective or, better, inter*-*corporeal existence*. This vulnerability not only defines the concrete, primarily and mostly unthematic prerequisite for our interactions. Instead, within these interactions, it is always at stake—be it in its cultural codifications or symbolic over-determinations.

With this in mind, it becomes increasingly clear that violence must be seen as a *quintessentially relational phenomenon*. Three major aspects of this assertion must now be stressed: firstly, violence must be considered in the light of the steadfast *relation between embodiment and symbolicity*. Secondly, violence must be examined as a specific *form of interaction with one’s own and, correlatively, the other’s vulnerability*. Thirdly, violence is an *event that occurs within the framework of its orders*, that is, it must be understood *in terms of the relational genesis of its differing shapes and forms*. Though it is beyond the scope of this essay to provide a comprehensive discussion of this overall relational constitution of the phenomenon, I will nevertheless give a number of pointers on how violence should be understood in this perspective.

The first is the already comprehensively expounded relation between embodiment and symbolicity. As I have shown before this is of fundamental importance if one is to grasp the other relations. The second one is the relational constitution of the subject-relative experience of our senses of violence, which derives its intelligibility exactly from its ground. This becomes intelligible in a passage from Pierre Bourdieu’s *Masculine Domination*, which clearly shows how violence can only be understood within the context of an essentially relational interaction with one’s own vulnerability and that of the other:

Like honour—or shame, its reverse side, which we know, in contrast to guilt, is felt *before others*—manliness must be validated by other men, in its reality as actual or potential violence, and certified by recognition of membership of the group of ‘real men’. A number of rites of institution, especially in educational or military milieux, include veritable tests of manliness oriented towards the reinforcement of male solidarity. Practices such as some gang rapes […] are designed to challenge those under test to prove before others their virility in its violent reality, in other words stripped of all devirilizing tenderness and gentleness of love, and they dramatically demonstrate the heteronomy of all affirmations of virility, their dependence on the judgement of the male group. […] Manliness, it can be seen, is an eminently *relational* notion, constructed in front of and for other men and against femininity, in a kind of *fear* of the female, firstly in oneself. (Bourdieu 2002: 52f.)

The quote clearly shows how the relevance of violence for active identification and the power of violence to subjectify its agents passively are intertwined. Our embodied existence is the medium and juncture of this intertwining and the ambiguities it entails. At least in our modern western and the westernized tradition, we are used to existing according to the ideals of personal autonomy and sovereign action. Viewed against this background, the quote makes clear how the phantasmatic figure of the “autonomous body” shapes (at least) the self-understanding of the modern subject (see Bergoffen 2003a). In this context, Bourdieu manages to highlight our “inner history of violence” in a lucid manner (Liebsch 2003: 42f.) as well as “our own deep traditions of violence” (Whitehead 2007: 50). His analysis shows that it is on these bases that we reject our weakness and vulnerability, but also project our fear thereof onto others (see Kearney 2003)—others who must therefore be “brought into line with violent means” or who must be “protected” as a result of a paternalistic logic of power (see Bergoffen 1990, 2003b; Miller 2002).

The third relation I mentioned is equally apparent in Bourdieu’s text. The *direct* violence described in there finds much of its motivation in daily rituals of (often normalized) violence, that is, in “the *violence of everyday life as multiple,* as *normative* (and normal), as the outcome of the interaction of changing cultural representations, social experience, and individual subjectivity” (Kleinman 2000: 238). In this passage, Kleinman presents nothing other than a socio-phenomenological rehabilitation of the concept of *structural* violence. It is based on his recourse to a constitutive interweavement of embodied existence and social orders of meaning through a “sociosomatic interconnection” of “narratized fate and an experienced agency,” in Kleinman’s words, that “interfuse the social body and the lived body” (2000: 239; see also Whitehead 2007: 49). It is crucial that Kleinman no longer defines *structural* (and also *symbolic* and *cultural*) violence in Galtung’s terms, that is, prescriptively as a lessening or impediment of potentialities (see Galtung 1969).^20^ Instead, given his (at least implicitly) socio-phenomenological approach, he analyzes these forms of violence descriptively—even though he does not quite formulate it in this way—in terms of selective and exclusive practices of sense-formation and institution that, to a large extent, take place in the back of our consciousness, i.e., in our embodiments.^21^

Instead of viewing violence, then, simply as a set of discrete events, which clearly could also be the case, the perspective I am advancing seeks to unearth those entrenched processes of ordering the social world and making (or realising) culture that themselves are forms of violence: violence that is multiple, mundane, and perhaps all the more fundamental since it is the hidden or secret violence out of which images of people are shaped, experiences of groups are coerced, and agency itself is engendered. Because the cultural prefiguring and normative social workings of violence shape its consequences as forms of suffering and means of coping, such violence must also be at work in the institutions that authorize response and in the ordinary practices of engagement. […] The violences of everyday life are what create the ‘existential’. In this view, the existential is not the result of a uniform human nature but rather emerges out of the inherent multiplicities, ironies, and instabilities of human conditions (shared and particular) in local moral worlds. (Kleinman 2000: 239)

Kleinman’s portrayal of the interweavement of different forms of violence shows the third relational structure of the phenomenon of violence in exemplary fashion. His analysis makes clear that a relational approach is required to understand the interplay of various phenomena of violence and its relevance for both the active formation of identity and the subjectifying, i.e., socio-technological implications this entails.

When seen as a whole, the consequences of the proposed relational approach are of significant importance to research on violence. The development of this approach, which places a *weak* subject at its center, might allow for the further development of an *integrative theory of violence*. An integrative approach, however, is significant in the following three ways: firstly, it allows one to think of violence in terms of embodiment *and* symbolicity, i.e., in terms of their relational genesis. Secondly, it makes it possible to bridge the “abyss,” which threatens to separate the apparently exclusive perspectives of those involved in violence. Thirdly, this approach opens up the possibility of providing proof that violence does indeed have different faces, each motivating the other. To further test this approach and to fathom the relational structure of violence *in concreto* must be the task of applied phenomenological analyses, whose possible scope I shall briefly discuss in the next, concluding section of this essay.

## Conclusion: Possible Applications and Future Lines of Argumentation

The analytical strength and practical feasibility of the outlined relational approach would need to be demonstrated via its application on a variety of phenomena of violence ranging from the many faces of interactive violence, over social violence, like, e.g., racism, to the difficult forms of organized, e.g., genocidal violence. As for simple reasons of space, I cannot provide such a demonstration here.^22^ Yet, to offer at least an exemplary outlook on the practical applicability of the approach, I will focus briefly on an exemplary case of interactive violence, the *slap in the face*.

This form of violence occurs in manifold contexts and is used for a variety of ends. A slap in the face is undoubtedly *physical* violence. However, it is precisely this attribution that is often refuted or rendered doubtful. You can hit somebody in the face with the fist in order to injure her or to knock him out (this may be the case in a random brawl but also in well-regulated martial arts, which declare a certain range of injuries to be legitimate but delegitimize others) or you may slap somebody with the open hand, i.e., give her a “clip round the ear”. Focusing here only on the latter, it is yet beyond doubt that even this so-called “minor violence” bears the potential to physically injure the beaten one^23^ and that, indeed, this is frequently its purpose. Whether or not it is corporally injurious depends upon the way it is “given” and upon the constitution of its “recipient”. In any case it seems to be essential that a slap in the face does not primarily (or only) intend a violation of the other person, but that it serves as a vehicle for other intentions. Yet, a slap in the face is never simply a “symbolic gift” but should rather be viewed as a symbolic counter-gift. In other words, it is through-and-through both an inter-corporeal and communicative action that aims to restore broken hierarchies or to create new orders. The economy implied in phrases like “to give a clip round the ear” or “to get a good beating”—you get something for something, in response, e.g., for a breach of regulations or misconduct—indicates that what is at stake here is not a form of *fight*: in a fight (relative) peers face each other, whereas a beating points to the violent actualization of pre-existent power relations. Given this, slapping can, e.g., be analyzed (a) as a means of “pedagogy” and “education,” (b) as a social ritual of dishonoring that calls for satisfaction (as in “codes of honor” and dueling) and (c) as a de-socializing practice, i.e., in terms of collective debasing and, finally, desecration, in the context of genocidal practices, e.g., in Nazism.

As for the context of *education*, I propose to draw upon Sartre’s analysis in his *Notebooks for an Ethics*. Sartre there shows clearly that the one who uses such violence, which is often embellished “as a form of affectionate devotion and pedagogical care for the immature being whose custody one has assumed” (Speitkamp 2010: 27), in the last analysis becomes “immoral [for ethics]” (Sartre 1992: 194) and has to turn into “pitiless stone” (Sartre 1976: 38). This clearly shows not only that a normative preconception of “physical violence” leads us to overlook “that such ‘minor violence,’ too, is per se a humiliation of its victim, that it demonstrates power, communicates helplessness, and signifies emotional violation. The symbolic dimension of violence and its connection with the honor and dignity also of an adolescent were lost to invisibility” (Speitkamp 2010: 52). Secondly, it points at the fact that such “minor violence” done in the name of an order (universal reason) against those deemed “unable to reason” leads to an *indifference vis*-*à*-*vis the pain and suffering of the other*. Thirdly, this insight should make us sensitive to the fact that this violence even appears to *appertain* to the apparently abnormal other:^24^ Given her otherness, she seems to require such treatment in order to finally find *admission* to this order once she has developed or is refined, purified, renewed, etc. Finally, it is of paramount importance to see that the use of such violence structurally predetermines and concretely shapes a habitus of indifference on the part of the “rationally” acting “autonomous subject” (see Speitkamp 2010: 24f.).

The second example, the understanding of the “clip round the ear” as a means to *dishonor*, concerns the so-called “culture of honor” (see Baldick 1965; Frevert 1995). With the related practice of “dueling” in order to “gain satisfaction” for an offense of one’s honor, we touch upon a heavily ritualized form of interactional violence. However, a closer look quickly reveals the underlying intrinsic violence of a social nexus that forces those under its sheltering spell to violently (and thus illegally) affirm their position (and, consequently, the existing structures of domination) or that, conversely, threatens to expel them from its safety. Here the necessity *to become habitually indifferent against one’s own suffering*, pain, and fear of death, which one has to endure in order to assure one’s own integrity and social agency, becomes palpable.^25^ In Collins’ words: “Behind the façade of honor and disrespect, the opponent to be overcome is always one’s own fears, and doing so makes one part of a death-defying ostentatious elite” (Collins 2008: 220). Here the socio-technological function of codes of honor and the violence they entail becomes visible:

An honor code is an ideology of stratification that emerges in particular kinds of social structures; it is a justification, a moralistic cover that operates to give a veneer of legitimacy to stratification, just like any other stratification system. In this case, it is the blatant stratification of the violent over the unviolent, of groups who make toughness an organizing principle over those who are not so tough. (Collins 2008: 233)

The third case I would like to mention concerns the desecrating role of the ritual of “minor violence” in German Nazism.^26^ In this context, Lindenberger and Lüdtke rightly underscore the visibility and efficacy of how (and to what extent) “everyday conventions of ‘minor violence’ are interrelated with codes of violent domination—through to the excesses of violence that embody the ‘breakdown of civilization’ in modernity” (Lindenberger and Lüdtke 1995: 23). It appears decisive to me that “clips round the ear” indeed “rendered speechless”; that they have remained “dumb” and underexposed for a long time in the historiography of the Holocaust, but that they have not at all been “mute” in themselves. To the contrary, this “minor violence” showed in a paradigmatic way that violence, which does not speak, yet functions in an essentially communicative way (Lorenz 2004: 20). Ilja Srubar has coined the syntagma “a-semiotic communication” in order to show that all violence, on the one hand, always appeals to an ultimate bodily reference (*leibhaftigen Letztbezug*), and that, on the other hand, it generates sense on a pre-linguistic level precisely by interrupting or breaking the process of semiosis, i.e., of making sense (see Srubar 2013). It is key, therefore, that the relative regularity of this “minor violence” does not exclude the embodied anticipation of an excessive intensification of violence, but rather conjures it up in its very possibility on a pre-discursive level:

At the same time, every clip round the ear entailed the possibility to lash out even more harshly, to add to the initial ‘minor’ violence. Clip round the ear were never final. The threat of additional violence could never be excluded. In German fascism this had consequences in two different ways: For those who were hit it became the threat of ‘more’. The breach of rules—not only adolescents were hit, but all, regardless of their age or their former social position—shows that it was not about strengthening a hitherto existing authority. A ‘New Germany’ became manifest also and especially in these deeds of ‘minor violence’. (Lindenberger and Lüdtke 1995: 26)

Thus, we doubtlessly have to regard this “minor violence” as a sign for something else and, hence, as a contribution to the “breakdown of civilization”. Indeed, its function was also to adjoin the “normality of familiar exclusions of others in a rather casual way with the killing machine” (Lindenberger and Lüdtke 1995: 27); further, given its apparent normality, to strip it of its spectacular character; and, finally, to convert *à la limite* all that was to follow into the normality of a pure (!) act of bureaucracy.

The key question, however, remains: how was it *possible* that the extermination “in its everydayness was run by human beings” (Lindenberger and Lüdtke 1995: 27)? To retrace this very possibility back to the incursion of irrationality or the “absolute evil” seems to deflect from the necessity and obligation to understand it. Therefore, I again deem it as the key aspect to focus on the *indifference* with which “minor violence” was not only committed in the everyday life-world, but, indeed, with which it was *produced* to an unimagined extent by exactly this violent practice. In this context, we should, in the last analysis, consider indifference in terms of the *embodied habitus* produced by the societal violence exerted by the project of total social integration: this violence not only renders the subject indifferent vis-à-vis *the suffering of others* but rapidly also vis-à-vis *itself*.^27^ These two processes of becoming indifferent aggravate each other up to the point where the execution of this violence appears as the *duty* of a split subject who, armed with this duty, becomes able to execute the “work of extermination” under the pretense of its normality.^28^

In the light of these examples it becomes clear that we have to understand the ciphers and codes of human vulnerability relationally with regard to the inner violent history of its proponents, i.e., in terms of their embodiment and subjectification. Interactive violence must, thus, not be analyzed independently of the embodied ciphers of autonomy and the functioning codes of violent domination which both pervade our cultural and political economies of social interaction. The acting subject, in other words, always finds itself already within a world shaped and ordered by manifold violence. This being the case, such an ordering function needs to be acknowledged as an integral part of our subjectivities, as a part inscribed in the flesh at the back of our consciousnesses. It has rightly been argued that “minor,” “everyday violence” (like slapping) mirrors “codes of violent domination” (see Lindenberger and Lüdtke 1995: 22–24). Yet, it is also of paramount importance to realize that the use of such violence shapes the habitus of the “rationally acting autonomous subject” of violence and, thus, predetermines its agency *on a pre*-*reflective level*. Viewed against the background of political modernity, this takes us to the *paternal* genesis of our habitus and to the corresponding images of the “masterful and autonomous body”^29^ that shape our sense of integrity and personal identity. Given that, we can see that “minor violence” not only mirrors such “codes of violent domination” but—by configuring our ideas of what it means to be an “autonomous” and “masterful” subject—rather serves as a medium for their implementation, extension, and even creation. Adopting a relational approach might thus help us to arrive at a deeper understanding of the silent interplay of our embodiment and its symbolic over-determinations that is of paramount importance both for the genesis and the dynamics of violence.

## Footnotes

1 On this critique of a one-sided fact-based analysis of violence, cf. Collins (2008: 2f.). It has also been shown that this type of analysis tends to favour a limited notion of violence, i.e., one restricted to physical violence, see von Trotha (1997), Nedelmann (1997), Schroer (2004).

2 This is not only my theory, but also the sociological demand recently made by Wieviorka (2007: 112). It is shared by recent German-language sociological work on violence and by so-called “ethnographies of experience” that deal with the topic at hand.

3 I prefer to use the here introduced terminology instead of notoriously imprecise nomenclature specifically of “victims” and “perpetrators,” since this introduces a legal order-determined perspective that does not do justice to the distinctions drawn by theories of social action and micro-sociological accounts (see Hitzler 1999).

4 As critically shown by the author of this article, this assumption is shared not only by system theory but also by social constructivism and a variety of other theoretical approaches in social philosophy (Koschorke 2008).

5 See, e.g., Sartre (2004: 716–734); on the violence of colonialism and racism as practice and process; compare De Warren (2004).

6 This finding applies to all those involved in violence. Thus the subjectification of violent actors, e.g., in the case of racism, causes their de-subjectification within the horizon of a norm that can impossibly be complied with (Yancy 2012; Staudigl 2012); the de-subjectification of recipients of collective violence causes their subjectification as members of the attacked group (ethnicity, etc.) (see Sartre 1976); the experiencing of violence by a witness, this specific type of *ethical* violence that she suffers, constitutes her as a witness, who must prove her new-found status by actively testifying to it, from an irretrievable distance and (possibly traumatic) passivity as regards the event (see Hatley 2000).

7 This is particularly so as concerns *orders of violence* inside of which a social praxis or event is branded “violent” or, indeed, where any such interpretation is outright rejected. It is this practice of attribution—a veritable event in the struggle for definitional mastery in such practices—that exemplarily exposes the violence that results from orders. This is because it attributes certain experiences to violence, while disassociating others from it. In particular, the lack of recognition for violence suffered is key since it can contribute to the supposedly legitimate unleashing of counter-violence.

8 This is the case with Hannah Arendt, whose *On Violence* denies power all violence, that is, it firmly assumes that violence from legitimated sources of power *can be sublated* (see Arendt 1970).

9 On the differentiation of the concept, see, helpfully and most recently, Behnke (2010).

10 The term “generative” is to address the irreducible combination of inter-subjectivity and historicity (see Steinbock 1995).

11 The problem of history only emerges upon the late Husserl’s admission that sense cannot simply be ascribed to the sense-bestowing intentions of the subject. This becomes evident when he states that history is nothing other than “the vital movement of the coexistence and interweaving of original formations and sedimentations of meaning” (Husserl 1970: 371). The later Merleau-Ponty, on his turn, concretely unfolded the implications of this insight in taking into account the chiasmatic intertwining of our embodiment and its symbolic articulations (see Merleau-Ponty 2010).

12 With the idea of an ethical resistance, I subscribe to one of the basic theses of Levinas, who, in the *face* of the other, sees a scheme at work that renders me ethically responsible for the other on a pre-normative and pre-intentional level (see, for instance, Levinas 1979: esp. 187–219).

13 I thus take on and modify Bernhard Waldenfels’ definition of violence as “injury, specifically of *demands*” of a “self-referential integrity” (Waldenfels 1991: 105; see 2003: 145f.). On the corresponding differentiation of “direct” or “irrupting violence” as an injury of the corporeal integrity of the subject, on the one hand, and “indirect” or “exclusionary violence” as an impediment to integration into an overarching (social, cultural, generative, etc.) context that has an integrative function or is considered to have one, on the other, see Delhom (2000).

14 This concept, which comes out of Jan Patocka’s theory of the “movements of human existence” (see Patocka 1991) seems to be particularly suitable to express adequately the phenomenological situation at issue here.

15 See Mensch (2009). In this article I consciously ignore the question of *linguistic* violence for reasons of space. Concerning this question the reader may refer to an ongoing and vigorous discussion (see Liebsch 2007; Herrmann/Kuch 2007; Krämer 2007; Posselt 2011). I also need to emphasize that the following sketch, which distinguishes between various forms of violence, does absolutely not pretend to be exhaustive: it is simply meant to indicate a variety of indirect violences that all exploit the symbolic co-constitution of human embodiment. A full-fledged account, which cannot be provided here, would indeed require a critical discussion with existing work on this topic, esp. Galtung’s seminal analyses on “structural” and “cultural violence” (see Galtung 1969, 1990).

16 See the “Appendix VI” to the *Crisis*, where Husserl famously addresses the so-called *Urstiftung* of geometry (Husserl 1970: 353–378). It must be added that Husserl’s term was first translated as “primal establishment” (1970: 72). Only later, following the translation of Merleau-Ponty’s works, especially his lecture courses, the translation “institution” became prevalent. A systematic elaboration of the concept can be found in the lecture course on “Institution and Passivity” (Merleau-Ponty 2010).

17 Though it cannot be done here, it would be appropriate in this context to connect Husserl’s and Merleau-Ponty’s notion of “habitual body” with Bourdieu’s concept of “habitus”.

18 This critique refers, of course, to Husserl’s theory of signification as outlined in his *Logical Investigations* and later also in his *Ideas* (see Husserl 1982: 295).

19 As I have already indicated, the “third party” ranges from witnesses to bystanders, onlookers, including followers. Generally viewed, the “third party” is not only important regarding the *situational dynamics* of violence (cf. Collins 2008: 19f. and *passim*). Especially the fact that questions concerning the objectivity and legitimacy of social events are raised with regards to her is of paramount importance.

20 Quite traditionally, Collins (2008) criticizes Bourdieu’s related concept of “symbolic violence” to “mudd[y] the analytical task” (2008: 24) and demotes it as “mere theoretical word play”. (2008: 25) Collins himself proposes to focus on the emotional “micro-situational processes” and “situational dynamics” that can lead to violence, i.e., fear, tension, and forward panic. This approach, however, does not at all contradict a theory of “symbolic violence” but rather requires one: this is due to the fact that “emotions” are not at all immanent qualities of interacting subjects but revolve around the interconnections of the social and the somatic, i.e., in the realm of inter-corporeal encounters and inter-kinesthetic affectivity that shapes our lived embodiment.

21 On this topic, see also Farmer (2004: 315), who also recurs to Bourdieu’s “habitus” in order to provide an analytic upgrade of the concept of structural violence: “Structural violence is structured and *structuring*. It constricts the agency of its victims. It tightens a physical noose around their necks, and this garrotting determines the way in which resources–food, medicine, even affection–are allocated and experienced. Socialization for scarcity is informed by a complex web of events and processes stretching far back in time and across continents”. It should be noted that Sartre’s *Critique of Dialectical Reason*—based on the notion of scarcity—bears much potential for the analysis of the relationality of structural and actual relations of violence, which has hitherto remained unnoticed in the socio-theoretical context.

22 For an extended analysis of the exemplary cases mentioned I refer to chapter 4 of my recent work on violence *Entwurf einer relationalen Phänomenologie der Gewalt* (forthcoming).

23 For medical evidence on this, esp. the „tin ear syndrome, “see Hanigan et al. 1987.

24 It should be noted here that “clips round the ear” are not only given to children, but have also been given to those who were said to be in need of “education,” like, e.g., housewives, farmhands, domestics, and, in a systematic way, the colonized people. It played and still plays also an important role in a variety of contexts as a “positive act of coming to age,” i.e., it paradoxically also figures to impart honor (see Speitkamp 2010: 28).

25 This is shown exemplarily in T. Fontane’s novel *Effi Briest*; see Speitkamp 2010: 7ff.

26 “In many testimonies of concentration camp survival […] not only were the beatings plentiful but also *clip round the ear*. Clip round the ear given by policemen, members of bureaucracy, train conductors, that is, not only by members of the SS. These thugs were persons who perhaps wanted to see themselves as ‘good Germans’—and were possibly former neighbors” (Lindenberger and Lüdtke 1995: 23). One can find various examples for the everyday practice of such “minor violence” in the Third Reich in Rosenstrauch, Pisarek, and Gross (1988) and Klemperer (1999). On “desecration” see, finally, Nahoum-Grappe (2002).

27 On this process of the violent formation of the armed male subject in German fascism see Theweleit (1987, 1989).

28 Regarding this concept of “hard work” and the necessity to work on one’s habitus see Himmler’s “Posener Rede” from 4 October 1943. In this context, it would be necessary to reflect upon the most diabolical violence inflicted by the Nazis, i.e., the instrumentalization of their victims for the execution of their “work”: what Primo Levi has termed the “gray zone” (Levi 1989: 22–51; see Agamben 1999: 21–26) is a realm that extends from the creation of the terrifying authority of the “Kapo,” who was entitled to do the initiating and daily beating of inmates and, thus, contributed to the systemic breakdown of “functioning trust” in the “social system” of the concentration camp, to the infernal work to be done by the *Sonderkommandos*.

29 I take this concept from Debra Bergoffen who has developed it in a series of articles (see Bergoffen 1990, 2003a, b).
